# Pre-exercise screening: role of the primary care physician

**DOI:** 10.1186/s13584-016-0089-0

**Published:** 2016-06-28

**Authors:** Elizabeth A. Joy, Linda S. Pescatello

**Affiliations:** Community Benefit Department, Intermountain Healthcare, 36 S. State St, 23rd floor, Salt Lake City, UT 84111 USA; Department of Kinesiology & Human Performance Laboratory, College of Agriculture, Health and Natural Resources, University of Connecticut, Storrs, Conneticut USA

## Abstract

Participation in regular physical activity is associated with a multitude of benefits including a reduction in chronic disease and premature mortality, and improved quality of life. All segments of society need to collaborate with one another in an effort to promote active lives.

The Israeli “Gymnasium Law” requires pre-exercise evaluation prior to exercise participation in a health club. Recently that law was modified to allow for participant pre-screening with the Physical Activity Readiness Questionnaire for Everyone (PAR-Q+). This change reflects the evidence that the risk of catastrophic events (e.g. heart attack) during moderate intensity physical activity is low, and the likelihood of detecting heart disease in asymptomatic adults is low. This change will likely reduce the number of individuals who require physician evaluation.

The American College of Sports Medicine (ACSM) recently updated their recommendations for pre-exercise evaluation. The ACSM guidelines have replaced risk factor assessment, with an algorithm that first stratifies based on current physical activity level, then by the presence of chronic disease, and/or signs and symptoms of chronic disease, and last by desired exercise intensity. The goal of these efforts is to reduce barriers to regular physical activity, by eliminating unnecessary medical evaluations. All adults should be encouraged to be physically active.

## Background

Physical inactivity is a global health problem that is vital to the primary prevention of over 35 chronic diseases [1, 2]. Moreover, exercise is an important treatment component for some of the most prevalent, costly, and deadly chronic diseases and health conditions in the world, including cardiovascular disease (CVD), diabetes mellitus, some forms of cancer, obesity, dyslipidemia, and hypertension, among others [3–6]. To achieve these health benefits adults are recommended to accumulate 150 min/wk of moderate intensity or 75 min/wk of vigorous intensity physical activity, or some combination of the two, on most, preferably all, days of the week [6]. Children and adolescents are advised to achieve 60 min of moderate to vigorous physical activity 7 days per week.

Shuval and colleagues surveyed college students in Israel and found that only 33 % of them met recommended levels of physical activity [7]. It will take a coordinated effort between multiple segments of society (e.g., schools, public health, healthcare) to address this public health problem.

The important role of healthcare providers in physical activity assessment and promotion is well established [8]. Physicians should assess the current physical activity level of their patients, along with a patient’s risks, readiness, and resources for participation in regular physical activity. A physician-performed pre-participation physical evaluation (PPE) is considered a standard, generally required, process for clearance to participate in competitive sports at many levels (e.g., high school, college, elite sport) [9]. Despite the required nature of this evaluation, there is limited/conflicting evidence as to the effectiveness of the PPE in identifying conditions that may place largely health adolescents and young adults, at risk for injury or illness as a result of sport participation [10].

Interestingly, the PPE process for adults participating in recreational sport or fitness activities is not generally required; yet adults, in contrast to children and adolescents, are at greater risk for complications and consequences related to an interaction among physical activity or exercise, chronic conditions, and orthopaedic limitations [11].

Contrary to the aforementioned observation that PPEs are seldom required for recreational adult athletes, a 1994 Israeli “Gymnasium Law” mandates that adults seeking to exercise in a health club receive a clearance physical evaluation prior to their participation in fitness activities at a club [12]. Recently the law was modified to allow those seeking health club participation, to self-complete the Physical Activity Readiness Questionnaire for Everyone (PAR-Q+) [13] in lieu of a physician evaluation [14]. It is estimated that 1 % of PAR-Q+ completers will require further evaluation by a physician prior to exercise participation [15]. While this modification will result in fewer physician consultations for preparticipation evaluation and clearance, there will still be adults directed to a physician for further evaluation. As such, it is important that physicians understand legal requirements, and are competent in performing PPEs for adults seeking clearance.

## Discussion

To better understand the current practices of Israeli physicians in performing adult recreational activity PPEs prior to fitness training in a health club, RD Hoffman, R Golan, and S Vinker [16] surveyed a convenience sample of Israeli Family Medicine physicians using an anonymous paper-based questionnaire. Respondents included physicians ranging from those still in residency training to more senior physicians – 135 of whom were included in the final analysis.

The questionnaire examined a number of constructs including: 1) the physician’s willingness to provide pre-exercise certificates, 2) actions taken by the physician prior to providing a pre-exercise certificate, 3) physician knowledge of their employer recommendations and regulations in providing the preparticipation service, and 4) the physician’s personal participation in weekly exercise.

The authors found that most respondents (63 %) were aware of guidelines for issuing pre-exercise certificates. Likewise, a majority (78 %) provided the certificate after performing a medical history and documenting it in the medical record. A similar percentage of physicians (77 %) would not issue a certificate to patients with heart disease, and would refer to a specialist for further evaluation and clearance. Consistent with prior research [17], physicians who exercise regularly were more likely to promote exercise and provide clearance to all of their patients (46 vs. 14.5 %) [16]. Finally, “exercising” physicians were less likely to order a cardiac stress test as a component of the clearance evaluation for cardiac patients.

The authors concluded that while virtually all of the physician respondents were willing to issue a pre-exercise certificate to most patients, there was heterogeneity in the pre-exercise evaluation. In addition, the new policy allowing for pre-exercise evaluation and clearance via response to the PAR-Q+ introduces new challenges. A single “yes” answer on the PAR-Q+ follow-up questions prompts recommendation to seek medical evaluation. When one considers the prevalence of chronic conditions such as prediabetes (an estimated global prevalence of >470 million by 2030) [18], high blood pressure (40 of adults 25 years of age and older worldwide) [19], and the percentage of US adults regularly taking at least one prescription medication (nearly 70 %) [20], it is possible that more than the reported 1 % of adults completing the PAR-Q+ will need physician clearance before participation in a health club.

Yet, the absolute and relative risk of exercise-related cardiac events in a general population of asymptomatic adults is low [21]. Exercise is safe for most people, and has many health and fitness benefits. In fact, the cardiovascular risk associated with exercise lessens as an individual becomes more physically active and fit. The evidence for pre-exercise screening as a strategy to prevent exercise-related cardiac morbidity and mortality is lacking [22], and at a minimum it serves as a potential barrier to adopting regular exercise while placing additional financial burden on individuals and the healthcare system.

In June 2014, the American College of Sports Medicine convened an expert panel to reevaluate the College’s pre-exercise screening recommendations [21]. The expert panel was composed of physicians and exercise scientists. Their charge was to examine the peer-reviewed literature, and propose a new evidence-informed model for exercise pre-participation health screening based on three factors: 1) the individual’s current level of physical activity, 2) the presence of signs or symptoms and/or known cardiovascular, metabolic or renal disease, and 3) desired exercise intensity. Of note, the new guidelines no longer include a cardiovascular disease risk factor profile as part of the decision making for referral to a healthcare provider prior to initiating a moderate-to-vigorous intensity exercise program. Likewise, the guidelines no longer recommend a low/moderate/high risk classification scheme.

The new American College of Sports Medicine guidelines make general recommendations for medical clearance versus recommendation for a specific set of medical exams or tests. The guidelines specifically state that the manner of clearance is left to the discretion of the healthcare provider. In support of this recommendation are the 2008 Physical Activity Guidelines for Americans that recommended that, “symptomatic persons or those with cardiovascular disease, diabetes, or other active chronic conditions who want to begin engaging in *vigorous* physical activity and who have not already developed a physical activity plan with their health care provider may wish to do so”, but does not mandate such medical contact.” [23] Nonetheless, if the healthcare provider performing the preparticipation physical evaluation (PPE) is looking for guidance on the medical examination or diagnostic tests they might consider, they can be referred to the *ACSM’s Guidelines for Exercise Testing and Prescription 9th Edition* [5].

**Fig. 1:**
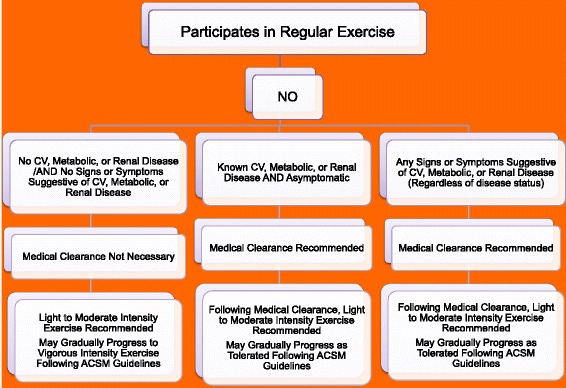
Prepar-cipa-on Clearance

**Fig. 2:**
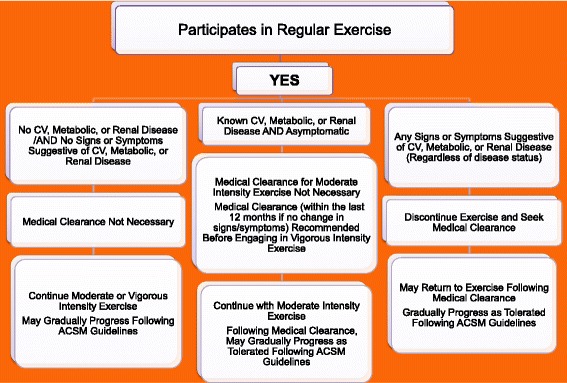
Prepar-cipa-on Clearance

The first decision point is whether or not the individual participates in regular exercise (defined as 3 or more 30 min sessions of moderate intensity physical activity each week). Figures 1 and 2 For the person For the person who does NOT participate in regular exercise, known cardiovascular, metabolic or renal disease, or signs and/or symptoms of disease should prompt medical clearance. However, for the person who is regularly physically active, the presence of known disease, albeit asymptomatic, does NOT prompt the need for medical clearance for moderate intensity physical activity. Medical clearance would be advised if there was a desire to increase exercise intensity to vigorous [21].

## Conclusions

The new American College of Sports Medicine exercise preparticipation health screening recommendations emphasize the important public health message of regular physical activity for all. They simplify the prescreening process by eliminating the need for medical clearance and/or exercise testing in many individuals, especially when low-to moderate intensity exercise is contemplated. The guidelines recognize that the hazards of exercise-related cardiovascular events are likely to be reduced by careful attention to a safe and effective exercise prescription.

The updated Israeli “Gymnasium Law” allows for pre-exercise screening with the PAR-Q+, as a strategy to detect conditions that may put health club participants at risk for exercise-related adverse consequences. Use of the PAR-Q+ will surely reduce the number of individuals seeking clearance by a physician; however given the global burden of chronic disease, physicians will still find themselves in a position to provide the required pre-exercise certificates. Given the lack of evidence for medical clearance and exercise testing as effective in mitigating exercise-related cardiac events, the responsibility of performing an appropriate history and examination lies with physicians providing care to patients seeking pre-exercise certificates.

The risk of sitting on a couch far outweighs the risk associated with moderate intensity exercise for the vast majority of adults. Efforts should be undertaken to facilitate participation in regular physical activity by all – including those with established disease. Guidelines serve as a foundation for clinical recommendations, but should not replace sound clinical judgment and shared decision-making between physician and patient.

## Abbreviations

ACSM, American College of Sports Medicine (Professional organization representing sports medicine, sports science and fitness professionals); CVD, Cardiovascular disease (Also known as coronary artery disease or ischemic heart disease); PAR-Q+, Physical Activity Readiness Questionnaire for Everyone (Questionnaire assessing readiness to participate in exercise); PPE, Preparticipation physical evaluation (Physical exam performed to assess health prior to sports or exercise participation)
